# Detection of illicit online sales of fentanyls via Twitter

**DOI:** 10.12688/f1000research.12914.1

**Published:** 2017-11-02

**Authors:** Tim K. Mackey, Janani Kalyanam

**Affiliations:** 1 Global Health Policy Institute, San Diego, CA, USA; 2 Division of Global Public Health, University of California, San Diego School of Medicine, San Diego, CA, 92093, USA; 3 Department of Anesthesiology, University of California San Diego School of Medicine, San Diego, CA, 92093, USA; 4 Department of Electrical and Computer Engineering, University of California, San Diego, San Diego, CA, 92093, USA

**Keywords:** fentanyl, prescription drug abuse, digital surveillance, fentanyl, counterfeit, Twitter, social media, internet pharmacies

## Abstract

A counterfeit fentanyl crisis is currently underway in the United States.  Counterfeit versions of commonly abused prescription drugs laced with fentanyl are being manufactured, distributed, and sold globally, leading to an increase in overdose and death in countries like the United States and Canada.  Despite concerns from the U.S. Drug Enforcement Agency regarding covert and overt sale of fentanyls online, no study has examined the role of the Internet and social media on fentanyl illegal marketing and direct-to-consumer access.  In response, this study collected and analyzed five months of Twitter data (from June-November 2015) filtered for the keyword “fentanyl” using Amazon Web Services.  We then analyzed 28,711 fentanyl-related tweets using text filtering and a machine learning approach called a Biterm Topic Model (BTM) to detect underlying latent patterns or “topics” present in the corpus of tweets.  Using this approach we detected a subset of 771 tweets marketing the sale of fentanyls online and then filtered this down to nine unique tweets containing hyperlinks to external websites.  Six hyperlinks were associated with online fentanyl classified ads, 2 with illicit online pharmacies, and 1 could not be classified due to traffic redirection.  Importantly, the one illicit online pharmacy detected was still accessible and offered the sale of fentanyls and other controlled substances direct-to-consumers with no prescription required at the time of publication of this study.   Overall, we detected a relatively small sample of Tweets promoting illegal online sale of fentanyls.  However, the detection of even a few online sellers represents a public health danger and a direct violation of law that demands further study.

## Introduction

A fentanyl crisis is currently underway in the United States, characterized by an influx of counterfeit fentanyl-laced prescription drugs (e.g. Xanax ^®^, Norco ^®^, OxyContin ^®^, and Oxycodone) now being advertised, sold and consumed by the public ^1^. The result of this counterfeit infiltration into the U.S. drug supply chain has been an alarming increase in fentanyl-related overdose, deaths and seizures, due to illicitly produced products containing undeclared fentanyls and related fentanyl analogues/variants (e.g. acetyl fentanyl, butyrfentanyl, and furanylfentanyl.) ^1– 3^

Fueling this public health emergency is a global network of fentanyl producers and traffickers including countries such as China (where powdered fentanyls and other synthetic opiates are sold with pill presses or sold as precursors used to manufacture fentanyl), Mexico (where fentanyls are manufactured in clandestine laboratories and smuggled into the USA) and Canada (where fentanyls are also manufactured and sold locally) ^1^. Unfortunately, criminals see an opportunity to sell cheap and deadly versions of fentanyl-laced pills to satiate demand driven by a national prescription opioid and heroin epidemic, with the U.S. Drug Enforcement Agency (DEA) estimating that a kilogram of fentanyl could generate $5–20 million in retail counterfeit sales ^1^.

Other studies have explored the public health consequences of counterfeit fentanyl and have argued for better surveillance and harm reduction approaches to curb consumption and demand ^2, 3^. However, the potential impact and easy accessibility of fentanyls sold direct-to-consumer via the Internet and social media has not been examined. Other studies have identified illegal marketing and direct sales of other prescription controlled substances by illicit online pharmacies and the use of popular social media sites to promote these services ^4– 10^.

No study has assessed the role of Internet in fentanyl product promotion, despite concerns from the DEA about documented covert and overt sale of fentanyls online ^1^. To better characterize these risks, we conducted a social media surveillance study using big data approaches to identify fentanyl promotion and sale via the popular microblogging platform Twitter. 

## Methods

Our data collection was part of a larger study exploring user behavior characteristics of illicit prescription drug abuse mediated by Twitter, with tweets collected over a five-month period (June–November 2015) filtered for the prescription drug abuse-related keywords, including the term “fentanyl.” ^5, 6, 10^ Data collection and analysis was carried out using a methodology combining Amazon Web Services (AWS) cloud computing virtual instances for data collection and machine learning algorithms to analyze tweets using both assisted and unassisted protocols used in previously published studies ^5, 6, 10^.

Specifically, we used a Biterm Topic Model (BTM) that can identify underlying latent patterns or “topics” present in a large corpus of tweets ^6, 11^. Given the input corpus of text, and a predefined number k, the model outputs a set of k latent topics present in the corpus. Each topic represents an underlying pattern associated with fentanyl twitter conversations that were then isolated and coded using human annotation. 

The number of topics to be detected was set to 40 and the alpha and beta parameters of BTM were set to 1 and 0.01 respectively. The output of the BTM was set to display the top 10 words with the highest weight for each topic. Each topic was manually reviewed based on these words and classified into “news” and “online pharmacy” themes (results discussed below). We also used a keyword filtering process using common terms included in the text of tweets associated with illegal online drug promotion, also known as “selling arguments,” (e.g. “buy”, “discount”, “price”) in conjunction with the BTM outputs. Code used in this study is available from https://github.com/kjanani/health_topicmodeling.

## Results

Our study collected 28,711 fentanyl-related tweets during 2015, a period when the fentanyl crisis was escalating ^6, 11^. The majority of topics detected in the whole corpus of fentanyl-related tweets were news-related (97.3%, n=27,940), detailing counterfeit fentanyl dangers reported in national and local media outlets.

After isolating Tweets related to news reports, we used a keyword filtering process in conjunction with machine learning to detect the subset of tweets associated with illegal online drug promotion. Using this approach, we detected 771 (<1% of total) tweets promoting the marketing and sale of fentanyls and other controlled substances online. These tweets were then manually annotated by the authors (inter-coder reliability kappa=0.98) to assess if they included a hyperlink enabling direct-to-consumer sale and purchase of illegal fentanyls. Nine unique tweets (not duplicates or retweets) and their associated hyperlinks were then identified for further analysis.

**Figure 1.**
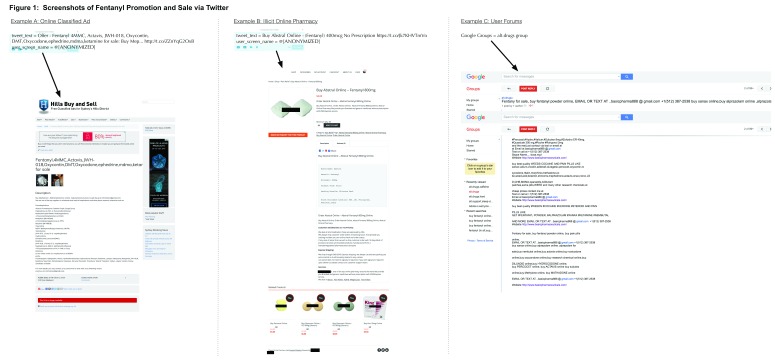
Screenshots of fentanyl promotion and sale via Twitter.

When examining website content of hyperlinks, 6 were associated with online classified ads, 2 with illicit online pharmacies, and 1 could not be classified due to traffic redirection. These results indicate some interesting trends. First, it appears that individual drug dealers use online classifieds ads to digitally advertise “street buys” of controlled substances (Example A, Figure 1). Additionally, one illicit online pharmacy that we detected and which is currently was still accessible at the time of initial publication of this study. offers the sale of fentanyls and other controlled substances direct-to-consumers with no prescription required (Example B). Its website purportedly offers Abstral® 800mg (fentanyl brand name) for $3.00 per tablet, and based on further inspection of WHOIS data, has its internet domain registration identity and location masked by an Internet privacy service company.

We note that there are certain limitations to this study. First the study only examined online promotion and availability at a single point-of-time, as websites of this nature are often removed or become inactive. Additionally, because of the illegal nature of the websites identified, we did not purchase fentanyls and test them for authenticity and potency. Buying controlled substances and making payments to an illicit online pharmacy raises serious legal concerns and is generally illegal.

## Discussion

Our results indicate that in our entire corpus of fentanyl-related tweets, direct-to-consumer sale of fentanyls occurred infrequently. This is not surprising, given the deadly nature and high potency of fentanyl, and that many victims may not actively seek its purchase ^1, 2^. However, the presence of even a few online sellers is concerning, as these actions represent a clear violation of Federal law (e.g. Controlled Substances Act and the Ryan Haight Online Pharmacy Consumer Protection Act) and directly endanger the public. Importantly, these sites may only be the tip of the iceberg, as hyperlinks in one result directed us to online user forums (Google Groups) actively selling fentanyl online (Example C). 

Our data collection occurred in late 2015, a period arguably at an early stage of the fentanyl crisis epidemiological curve, which may limit the generalizability of the results. Hence, this indicates that more research is needed to accurately characterize the dangers of the online environment in relation to counterfeit fentanyls. This includes longer term follow-up studies examining online promotion and access to fentanyl and interrogating other online platforms (including other social media sites, chat rooms/user forums, the deep web, and private sales facilitated by online and mobile technology).

## Data availability

The data referenced by this article are under copyright with the following copyright statement: Copyright: © 2017 Mackey TK and Kalyanam J

Raw datasets have not been made available per concerns regarding user information and confidentiality of publicly available processed data. This data is stored at the Global Health Policy Institute and is available upon request in a de-identified and aggregated dataset. Please contact the institute’s general contact email ( ghpolicyinstitute@gmail.com) for further information.

The code for BTM can be found at https://github.com/kjanani/health_topicmodeling.

Archived code: https://doi.org/10.5281/zenodo.1038177 ^12^

## References

[1] DEA: Counterfeit Prescription Pills Containing Fentanyls: A Global Threat [Internet]. dea.gov.2016; [cited 2017 Feb 17]. Reference Source

[2] Green TC Gilbert M: Counterfeit Medications and Fentanyl. *JAMA Intern Med.*American Medical Association,2016;176(10):1555–7. 10.1001/jamainternmed.2016.4310 27533891

[3] Frank RG Pollack HA: Addressing the Fentanyl Threat to Public Health. *N Engl J Med.* 2017;376(7):605–7. 10.1056/NEJMp1615145 28199808

[4] Mackey TK Liang BA Strathdee SA: Digital social media, youth, and nonmedical use of prescription drugs: the need for reform. *J Med Internet Res.* 2013;15(7):e143. 10.2196/jmir.2464 23892156 PMC3742396

[5] Katsuki T Mackey TK Cuomo R: Establishing a Link Between Prescription Drug Abuse and Illicit Online Pharmacies: Analysis of Twitter Data. *J Med Internet Res.* 2015;17(12):e280. 10.2196/jmir.5144 26677966 PMC4704982

[6] Kalyanam J Katsuki T Lanckriet G: Exploring trends of nonmedical use of prescription drugs and polydrug abuse in the Twittersphere using unsupervised machine learning. *Addict Behav.* 2017;65:289–95. 10.1016/j.addbeh.2016.08.019 27568339

[7] Raine C Webb DJ Maxwell SR: The availability of prescription-only analgesics purchased from the internet in the UK. *Br J Clin Pharmacol.*Blackwell Publishing Ltd,2009;67(2):250–4. 10.1111/j.1365-2125.2008.03343.x 19154446 PMC2670383

[8] Forman RF Block LG: The Marketing of Opioid Medications without Prescription over the Internet. *J Public Policy Mark.* 2006;25:133–46. 10.1509/jppm.25.2.133

[9] U S Government Accountability Office HGG: INTERNET PHARMACIES: Federal Agencies and States Face Challenges Combating Rogue Sites, Particularly Those Abroad [Internet]. gao.gov.2013, [cited 2016 Jan 11]. Reference Source

[10] Mackey TK Kalyanam J Katsuki T: Machine Learning to Detect Prescription Opioid Abuse Promotion and Access via Twitter. *Am J Public Health*: published online before print October 19,2017.e1–e6. 10.2105/AJPH.2017.303994 29048960 PMC5678375

[11] Yan X Guo J Lan Y: A biterm topic model for short texts. *The 22nd international conference.*New York, New York, USA: ACM,2013;12 10.1145/2488388.2488514

[12] Mackey T Kalyanam J: Detection of Illicit Online Sales of Fentanyls via Twitter. *Zenodo.* 2017 Data Source 10.12688/f1000research.12914.1 PMC5728221 29259769

