# Supplementary material for: Detection of illicit online sales of fentanyls via Twitter
Source: F1000Res. 2017 Nov 2;6:1937. [Version 1] doi: 10.12688/f1000research.12914.1 (PMC5728221; doi:10.12688/f1000research.12914.1)
Supplement: Peer review history

# Peer review: Referee response for version 1

**DOI:** 10.5256/f1000research.14002.r28806

**Published:** 2017-12-11

**Authors:** Róbert G. Vida

**Affiliations:** 1 Department of Pharmaceutics and Central Clinical Pharmacy, Faculty of Pharmacy, University of Pécs, Pécs, Hungary

An up-to-date and innovative methodological approach of a possible global problem of illegal trade of pharmaceuticals via the Internet is absolutely useful. The main messages are in accordance with the scientific literature.

 The concern highlighted by the Dr. Marvin D. Shepherd regarding the dissonance between the introduction and the other parts of the publication is real and similarly I suggest the reconsideration and rewrite of that part. Also it would be advisable to include the comparison of the legal and illicit use and highlight the sources of fentanyl products or fentanyl containing products (outpatient and inpatient settings). Beside the counterfeit fentanyl crisis another possible public health danger is hitting the health care system of the United States is the shortage of multiple drugs including opioid drugs as well (e.g.: fentanyl) necessitate the use of alternative supply channels (e.g.: importation of drugs outside the U.S., internet pharmacies) make the health sector more susceptible to illegal and counterfeit products.

 Beside the network science the Big Data type of analysis is a novel and hopefully in the future a routinely used methodology to identify and monitor illegal vendors and their connections in the field of illegal internet sale of pharmaceuticals. The more detailed description of the method or even the representation with a figure would help the understanding of it.

 The characterisation (e.g.: ordering is possible, LegitScript or VIPPS NABP legitimacy, products being sold, prescription requirements) of the identified online pharmacies would have highlighted the illegal internet sale of fentanyl products more accurately.

 Moreover the inclusion of comparison of the results with the literature data regarding other social media platforms or the simple google search methodology would give a more complete picture of this public health danger.

I have read this submission. I believe that I have an appropriate level of expertise to confirm that it is of an acceptable scientific standard.

# Peer review: Referee response for version 1

**DOI:** 10.5256/f1000research.14002.r27555

**Published:** 2017-11-30

**Authors:** Marvin D. Shepherd

**Affiliations:** 1 Division of Health Outcomes and Pharmacy Practice, College of Pharmacy, The University of Texas at Austin, Austin, TX, USA

My main concern is the discontinuity between the introduction and the research conducted. The introduction focused on problems of pharmaceutical purchased which were laced with counterfeit fentanyl. This is true and is a major health hazard as pointed out by the authors. Fentanyls are a deadly pharmaceuticals and again this was emphasized by the authors. However, the paper's research focus is on tweets from people who are interested in purchasing fentanyl illegally. It wasn't on the counterfeit fentanyl laced products. I suggest that the intro be rewritten and I mention the legitimate uses of fentanyl. Please mention that one of fentanyl's major uses is as a pain reliever in hospital surgical suites and must be carefully controlled by medical practitioners. To reiterate, the introduction doesn't match well the research content.

 The methodology approach was was appropriate and innovative. I know they provided a link for a full description of the search terms, but in reading it I suggest that they provided at least a partial list of the names used i.e. different spellings of fentanyl, different names for the same product (foreign names). Searching tweets was original, but it didn't produce huge number of tweets. **This is worth reporting in that few people tweet to purchase fentanyl**. The first thing I thought of was why tweet when you can do a google search and get right to the web sites? **This needs to be put in the discussion as to why the results were so low. ** I am sure there are other methods to find the product.

I have read this submission. I believe that I have an appropriate level of expertise to confirm that it is of an acceptable scientific standard.
